# Leveraging traffic citations as teachable moments: parent perspectives on a post-citation teen driving intervention

**DOI:** 10.3389/fpubh.2026.1794119

**Published:** 2026-03-23

**Authors:** Archana Kaur, Hannah Schneider, Dominique M. Rose, Cara Hamann, Corinne Peek-Asa, Jingzhen Yang

**Affiliations:** 1 Center for Injury Research and Policy, Abigail Wexner Research Institute at Nationwide Children's Hospital, Columbus, OH, United States; 2 Department of Epidemiology, University of Iowa, Iowa City, IA, United States; 3 University of Iowa Injury Prevention Research Center, University of Iowa, Iowa City, IA, United States; 4 Office of Research Affairs, University of California at San Diego, San Diego, CA, United States; 5 Department of Pediatrics, College of Medicine, The Ohio State University, Columbus, OH, United States

**Keywords:** driving feedback technology, parent-teen communication, teachable moment, teen driver, traffic violation

## Abstract

**Introduction:**

Teens cited for traffic violations represent a high-risk driving population, and traffic citations may function as psychologically salient teachable moments for behavior change. This study aimed to explore how parents interpreted and responded to their teen’s citation and how participation in a parent-focused, technology-supported intervention influenced parental engagement in promoting safe driving amid persistent teen crash risk.

**Methods:**

We conducted 38 semi-structured virtual interviews with parents of teens who participated in the intervention arms of ProjectDRIVE, a post-citation randomized controlled trial recruiting parent–teen dyads from juvenile traffic courts. Interviews explored parents’ reactions to the citation, experiences with intervention components, and recommendations for improvement and scale-up. All interviews were audio-recorded, transcribed verbatim, and analyzed thematically using an iterative coding process in ATLAS.ti.

**Results:**

Three themes emerged. (1) Parents’ reactions to the citation: Parents initially reported frustration, concern, or stress but often reframed the citation as a teachable moment to reinforce responsibility, accountability, and safer decision-making. (2) Parents’ experiences with ProjectDRIVE: Objective in-vehicle driving feedback increased teens’ awareness of risky behaviors and served as an external reference point, while structured parent communication strategies supported more open, less defensive conversations about driving safety. (3) Strategies for strengthening and scaling the program: Parents emphasized the importance of usability, relational support, and integration within existing systems, including juvenile courts, schools, and driver education programs, to enhance reach and sustainability.

**Conclusion:**

Traffic citations can serve as critical teachable moments for families of high-risk teen drivers. ProjectDRIVE supported this process by strengthening parent–teen communication, increasing accountability, and sustaining parental involvement during the early licensure period. Parent-focused interventions that integrate behavioral and technological strategies may offer a scalable, public health–oriented approach to reducing crash risk and promoting safer communities.

## 1 Introduction

Road traffic crashes remain a major global public health challenge, causing millions of deaths and injuries each year and placing a substantial burden on health systems, families, and communities worldwide. Teen drivers are disproportionately affected, with motor vehicle collisions (MVCs) consistently ranking among the leading causes of death in this age group (1–3). Despite progress through graduated driver licensing (GDL) systems and other regulatory and infrastructural measures, crash risk remains especially high during the early months of independent driving, when inexperience, developmental factors, and psychosocial influences converge (4, 5). These persistent risks highlight the need for innovative, multi-level approaches to road safety.

Teens who receive traffic citations represent a particularly high-risk subgroup. Traffic violations are strongly associated with subsequent risky driving behaviors, repeat citations, and increased crash involvement (6–10). From a public health perspective, a traffic citation is not only a legal event but also a psychologically salient stressor that may heighten risk perception, disrupt habitual behaviors, and prompt reflection by both teens and their families (11, 12). Such events may create a critical window for intervention, a “teachable moment,” during which teens and parents may be more receptive to guidance, support, and behavior change strategies (13–15). However, little is known about how parents interpret and respond to their teens’ citation or how this experience can be leveraged to promote safer driving.

Parents play a central role in shaping teens’ driving behaviors through supervision, rule-setting, modeling, and ongoing communication (16–22). Parental involvement is associated with reduced risky driving and crash risk, yet it often declines once teens begin driving independently (20–24). Following a traffic citation, particularly one processed through juvenile traffic courts that emphasize rehabilitation rather than punishment, parents may be uniquely positioned to re-engage in their teen’s driving and reinforce safety expectations (25–27). Whether parents view the citation as a failure, a learning opportunity, or a catalyst for growth may influence whether teens respond defensively or engage in meaningful behavior change. Understanding these parental perspectives is therefore critical for designing effective interventions during this high-risk period.

ProjectDRIVE is a parent-based randomized controlled trial (RCT) designed to support families following a teen’s traffic citation (28). The two-component intervention combines in-vehicle driving feedback technology with structured parent communication training. The driving feedback component included (1) real-time audio alerts triggered when vehicle movement exceeded preset g-force thresholds (hard braking ≤−0.45 g; sudden acceleration >0.35 g) or when speed exceeded 75 mph; (2) end-of-trip push notifications; (3) access to cumulative driving data; and (4) customized bi-weekly driving summary reports. The parent communication component, Steering Teens Safe (STS), was grounded in motivational interviewing principles and equipped parents with skills to engage teens in constructive, non-confrontational conversations about driving safety (28). Parent-teen dyads were recruited from juvenile traffic courts and randomly assigned to one of three arms: (1) Control (inactive feedback), (2) Driving Feedback Only (teens received feedback), or (3) Driving Feedback plus Parent Communication Training (teens received feedback and parents had access to driving reports and received communication training). All enrolled dyads were followed for 6 months, including 3 months of active intervention (28). Although parent-focused and technology-assisted interventions have shown promise in reducing risky driving, little is known about how parents experience these programs after a citation or how such interventions can be strengthened and scaled within existing systems (20–24).

Qualitative inquiry is well-suited to address these gaps by capturing parents’ lived experiences, interpretations, and recommendations. Parents’ perspectives can inform intervention refinement as well as broader implementation strategies, including integration with juvenile courts, schools, driver education programs, and insurance systems. Such insights are essential for advancing sustainable, system-level approaches to road safety that align with public health goals and the Sustainable Development Goals, particularly SDG 3 (Good Health and Well-being) and SDG 11 (Sustainable Cities and Communities).

Accordingly, this qualitative study explored three interrelated aims: (1) how parents reacted to and interpreted their teen’s traffic citation; (2) how they experienced participation in ProjectDRIVE and its components, including driving feedback and communication strategies; and (3) parents’ perspectives on how the program could be strengthened, scaled, and integrated into existing systems. By examining how parents transform a traffic citation into a teachable moment and engage with an intervention that combines technology with parent communication strategies, this study contributes to a broader understanding of how behavioral and technological approaches can be harmonized to reduce crash risk among high-risk teen drivers and promote safer communities.

## 2 Methods

### 2.1 Study design and participants

We conducted a semi-structured qualitative interview study to explore parents’/legal guardians’ (‘parents’) experience with their teen’s traffic citation and their participation in ProjectDRIVE. Participants were drawn from the two intervention arms (Driving Feedback Only, and Driving Feedback plus Parent Communication Training) of ProjectDRIVE, an RCT described above and in detail elsewhere (28). Eligible parent-teen dyads for ProjectDRIVE were parents whose teen (16–17 years) received a traffic citation. Teens were required to hold a valid license, have access to a vehicle and smartphone, and attend their juvenile traffic court hearing. For the qualitative study, parents were eligible if they were assigned to one of the two intervention groups, completed the ProjectDRIVE study, and consented to be contacted for an interview. Of 91 eligible parents contacted, 38 (42%) completed a virtual interview. This study was approved by the Institutional Review Board (IRB17-00318). This manuscript followed the Standards for Reporting Qualitative Research reporting guidelines (29).

### 2.2 Interview guide

The semi-structured interview guide was informed by the RE-AIM evaluation framework (30), the Consolidated Framework for Implementation Research (CFIR) (31), and existing literature on parental influence on teen driving safety (16–24), and implementation science (32).

For this study, we analyzed interview questions aligned primarily with CFIR domains, including:

- *Characteristics of Individuals,* reflecting parents’ reactions, motivations, and actions (e.g., “What was your reaction to your teen’s citation?” “What motivated you to be involved when your teen was learning to drive?” “What actions did you take to make this a teachable moment?”);
- *Intervention Characteristics*, focused on perceptions of ProjectDRIVE components (e.g., “What component did you enjoy the most and least?” “How was the program useful in helping you communicate about safe driving?”);
- *Inner Setting,* capturing family roles and dynamics (e.g., “What is your relationship to the teen who participated in ProjectDRIVE?” “Who else has played a critical role in your teen’s safe driving?”);
- *Outer Setting,* addressing the juvenile traffic court context and participation drivers (e.g., “What benefits do you see in juvenile traffic courts offering a parent-engaged program?” “If ProjectDRIVE was offered within the courts, what would persuade teens and parents to participate?”);
- *Process,* examining barriers, engagement, and recommendations (e.g., “What barriers prevent teens and parents from participating?” “What strategies could improve the program so you felt more prepared to apply these strategies?”).

Several questions also aligned with RE-AIM domains of *reach* (e.g., juvenile traffic court buy-in), *effectiveness* (e.g., perceived communication improvements), *adoption* (e.g., motivation to be involved), and *implementation* (e.g., barriers to participation). Overall, interviews explored parents’ reactions to the citation, their experiences with ProjectDRIVE and its components (e.g., driving feedback and communication training), their ongoing role in promoting safe driving, and recommendations for program refinement, scale-up, and system integration. The guide was reviewed by experts in teen driving safety and qualitative research and pilot-tested prior to use.

### 2.3 Study procedures and data collection

Thirty-eight semi-structured virtual interviews were conducted by trained interviewers (AK and DR) via Microsoft Teams between February 2022 and March 2025. Verbal consent was obtained, and participants were assigned unique identification numbers to ensure confidentiality. Interviews lasted about 45 min and were audio-recorded, then professionally transcribed verbatim as they were completed. The transcripts were reviewed by a researcher (AK or DR) and uploaded into ATLAS.ti (Version 25), a qualitative data software, for data coding and analysis.

### 2.4 Data coding and analysis

An initial codebook was created based on the interview guide. Two researchers (AK and HS) independently coded three transcripts to pilot the codebook, after which, the research team (AK, HS, DR, and JG) reviewed the initial coding, resolved discrepancies, and refined code definitions. The revised codebook was then applied to recode the initial transcripts.

Using the finalized codebook with clearly defined codes, two researchers (AK and HS) independently coded the remaining transcripts in ATLAS.ti. All transcripts were double coded to enhance accuracy and reliability. Coding discrepancies were discussed and resolved during regular meetings to ensure consistency and intercoder reliability. A thematic approach was used to identify the themes and subthemes, combining inductive coding with concepts from existing literature (33). Bi-weekly team meetings were held to refine themes and interpretations. Thematic saturation was achieved through identifying recurring patterns, an ongoing comparison of new data to existing codes, and continuously analyzing data until no new insights or themes were identified.

## 3 Results

### 3.1 Participant characteristics

Of the 38 parents interviewed, most were female (82%), White (82%), and held a 4-year college degree or higher (84%) (Table 1). Two-thirds were married (66%), and most reported a household income of $80,000 or more (74%). The average parent age was 51 years, and 66% worked full time. Most participants were enrolled in the Driving Feedback plus Parent Training group (68%). Slightly more than half participated with a son (55%), and teens averaged 16.6 years of age at the time of their citation.

**Table 1:** Demographics of study participants.

ID	Parent demographics		
	Dyad	Age	Marital status
P01	Mother–daughter	<50	Married
P02	Father–son	≥50	Married
P03	Father–daughter	≥50	Married
P04	Mother–daughter	<50	Other
P05	Mother–daughter	≥50	Other
P06	Mother–son	<50	Married
P07	Mother–son	<50	Other
P08	Mother–daughter	≥50	Other
P09	Mother–daughter	≥50	Married
P10	Mother–son	≥50	Other
P11	Mother–son	≥50	Married
P12	Mother–daughter	<50	Married
P13	Mother–son	<50	Other
P14	Mother–daughter	<50	Married
P15	Mother–son	≥50	Other
P16	Mother–son	<50	Other
P17	Mother–daughter	≥50	Married
P18	Mother–daughter	<50	Other
P19	Father–son	≥50	Married
P20	Father–daughter	<50	Married
P21	Mother–daughter	≥50	Married
P22	Mother–son	≥50	Married
P23	Mother–daughter	≥50	Other
P24	Mother–daughter	≥50	Married
P25	Father–daughter	≥50	Married
P26	Father–son	≥50	Married
P27	Mother–son	<50	Other
P28	Mother–daughter	≥50	Married
P29	Mother–son	≥50	Married
P30	Mother–daughter	<50	Married
P31	Mother–son	≥50	Married
P32	Mother–son	≥50	Married
P33	Mother–son	≥50	Other
P34	Father–son	<50	Married
P35	Mother–son	<50	Married
P36	Mother–son	≥50	Married
P37	Mother–son	≥50	Married
P38	Mother–daughter	<50	Other

### 3.2 Themes and subthemes

Three major themes were identified from the interviews: (1) parents’ reactions to their teen’s citation, (2) parents’ experiences with ProjectDRIVE, and (3) strategies for strengthening and scaling ProjectDRIVE (Table 2).

**Table 2:** Parent interview themes, subthemes, and quotations.

Theme One – Parents’ reactions to their teen’s citation	
Subtheme	Quotation
1a. Initial response	*“I was at work, and I grabbed my keys and sat down [whatever] was in my hand and said, I have to go because X [has] been in a car accident…and tried to keep him calm and he was actually pretty mature about it, so I was pleased about that.” P10, mother*
	*“At first I was angry…But it was a life learning lesson for her too.” P21, mother*
	*“It scared me with him speeding and flying around semis…We need some kind of reality check on how dangerous that can be.” P07, mother*
1b. Using the citation as a teachable moment	*“We talked about being a defensive driver, driving not just your car, but paying attention to the cars that are around you… [it] scared her for a little while at the beginning. So she did not want to drive… but I’m not that type of mom. You’re going to get back out there and drive because I’m not going to keep driving you around.” P18, mother*
	“*She recognized, I do not want this to happen to me again So what do I need to do? And just finding the solution instead of just focusing on the problem.” P04, mother*
	*“I took the keys away…his car, he was restricted to certain hours…I’ve been monitoring him like a hawk still kind of do.” P16, mother*
1c. Perceived impact	*“We made her pay for the fine, all the costs, cost associated with it…*.*Her license was suspended for I think it was six months…And we explained to her, I’m writing the check, but this money is coming out of your account.” P01, mother*
	*“He’s definitely more aware of his surroundings. Now, in all honesty, that could have happened anyway because of the citation… but I think he’s also a little more careful with his braking.” P33, mother*
	*“No, and I was happy he got it actually, because I think it helped. I think nobody was hurt, let us just let this hopefully be a lesson and just do not be stupid.” P32, mother*

Theme Two – Parents’ experience with ProjectDRIVE	
Subtheme	Quotation
2a. Perceived benefits	*“My experience with the project was pretty good. I liked some of the helpful information that they had on the website that I went over and reviewed with [my teen/interventionist]. I think my [teen] having the device installed into her car gave her a good idea how often she speeds and how she stops and how good she follows the rules. It also provided me with further insight.” P12, mother*
	*“The modules were good on the web, pointing out the different things to be aware of and talk about. I thought those were pretty good just because some of them you do not come across too often, so that’s a nice reminder like, “Okay, think about this” kind of thing.” P28, mother*
	*“I feel like it circled back and brought a little more attention to some very specific things in driving that I do not know that she got from her driving class… But when she was doing those weekly [activities] and I had to do [them] too, I would kind of, every once in a while, I’d be like, Hey, did you do that? …I try to break things up as a teacher….” P20, father*
	*“[Teen] was cool, like, “Okay. Yeah, I was speeding.” I’m like, “Hey, what’s going on? What happened?” “Oh, I was leaving soon, and I had to get here.” And we have conversations like…. “Well, you have got to make better decisions,” …You just reinforce…” P34, father*
2b. Opportunity for ongoing communication	*“I enjoyed the one-on-one with the recorded conversations. I think that was good. And just clueing me in to slow down and try to talk to him. He′s got his license, he’s got some freedom, so you got to talk to him as if they are mature enough to handle that freedom.” P29, mother*
	*“Probably what I liked the most was kind of what we have already talked about was it set up some conversations that we may not have otherwise had, so I thought that was good. It kind of forced that level of communication.” P25, father*
	*“Once they are driving on their own, and they have been doing it for a while, then the need to have conversations about does not come up unless it’s the first snowstorm of the year… So it did make us kind of stop and talk about things that we might not have otherwise” P22, mother*
2c. Objective driving feedback	*“He was surprised when he looked at some of the reports at how many quick stops the device was registering. It’s like you do not know what you do not know and I do not think teenagers are quite as self-aware as adults are, obviously, but I do think that was beneficial” P36, mother*
	*“I just think having that device in the car forces them to drive safely and then maybe driving safely under that condition, the duration of the study.” P14, mother*
	*“Paying attention to the weekly reports…I think that’s where he can see it right in front of him. You had 30 hard stops in a month. I think the results are right there. They can see evidence right in front of them and that’s what gets their attention right away.” P02, father*

Theme Three – Strategies for strengthening and scaling ProjectDRIVE	
Subtheme	Quotation
3a. Increasing adoption and stakeholder buy-in	*“If there’s data to show these teens… did not get another citation… showing just the data of this is how they are driving now, get them to buy [in] would be good” P35, mother*
	*“I know that the judge here in Delaware County is a very personable guy and very involved in the community, and maybe everybody’s not, but I’m certain that he talks with other judges in the area. If you had an advocate of a judge that said, ‘I’m instituting this [ProjectDRIVE],’…” P30, mother*
	*“The only thing that the judge said we should try this, and it was a really good program, so his endorsement was positive enough for me. So, if you have someone of that level saying This is legitimate and good” P08, mother*
	*“If you can show that it does reduce the recidivism there, possibly a good student discount for insurance, because we are all about our kids, but we know it takes money and that’s the first thing that you spend…” P26, father*
3b. Integrating the program into existing systems	*“I feel as though this was a good study that could be utilized in driving courses to a lot of the driver ed programs that this could be a good addition to those as well” P18, mother*
	*“I think if you could really get this involved with a court, and it’s more structured and there’s more of an outcome that if you do not do this, I think then you could get better outcomes.” P23, mother*
	*“Make more people take it, make it part of the teen driving course… I think it was a really good program. One of our friends happened to have had a similar car accident, and so I knew about the program when we went to the class because he had been asked to be in it.” P10, mother*
3c. Improving program navigation and relational engagement	*“Streamline it, like the app…. I cannot do so many websites. And then there was one website that had two different entries. That was super confusing.” P27, mother*
	*“If you can get that little thing to say something instead of a beep, because if it actually said, ‘Hey, you are speeding,’ or ‘Hey, you are…’” P28, mother*
	*“I think it’s extremely valuable… I just think the accountability of someone else inserting themself into the situation and saying, this is a serious offense. You do not want to be in this position and here are the reasons why. And it just felt like you had someone linking arms with you as you walk through that process with your team.” P17, mother*

#### 3.2.1 Theme 1: Parents’ reactions to their teen’s citation

This theme captured how parents initially responded to the citation, how they used the event as a teaching opportunity, and how they perceived its impact on their teen’s driving. Three subthemes emerged, including initial response, using the citation as a teachable moment, and perceived impact.

#### 3.2.2 Subtheme 1: initial response

Parents commonly described feeling angry, shocked, or upset when learning about the citation. Yet many balanced these emotions with efforts to support their teens by responding with empathy and creating a sense of safety. One mother described how she remained calm to keep her daughter composed, *“…I had to get her calmed down so that she could go to practice… I did not want her to get so flustered…and so I just talked her through it”* (P01, mother). While all parents expressed relief that their teen was safe and unharmed, some also voiced frustration with their teen’s judgement: *“I was mad… like any parent, you are upset. I felt awful because X was literally sobbing, and he felt terrible… he was literally missing his final exam too. It was super stressful”* (P31, mother). A few parents imposed immediate consequences, such as temporary loss of driving privileges. As one noted, *“And we were disappointed….We took his driving privileges away for a short period of time”* (P19, father). Others emphasized the real-world implications, such as insurance increases, *“I told her about the importance of safety, about how her insurance prices increased…”* (P12, mother). Overall, parents stressed the importance of balancing consequences with support to help their teens become more responsible drivers.

#### 3.2.3 Subtheme 2: using citation as a teachable moment

Parents frequently reframed the citation as an opportunity for learning rather than punishment. Many saw the citation as a teachable moment for growth and used the incident to initiate conversations about the responsibilities that come with independent driving. As one parent explained, *“The idea being that he’s got a really serious responsibility as a driver and….until he was able to prove that he was more responsible, then we were able to liberate his driving more…”* (P15, mother). Parents also emphasized accountability, often through financial consequences. One noted, *“I made him pay the cost for the ticket. He wasn’t working…he wrote me out a payment plan with due dates…”* (P13, mother) and another shared, *“When she pays her insurance every month and she’s mad that it’s so expensive, I’m like, ‘Well, you learned a very hard lesson.’”* (P38, mother). Some parents reflected on their role in shaping their teen’s driving behaviors. One parent noted, *“Put some responsibility on the person who taught them to drive. So that I think that kind of made me feel like maybe I should have taught him something differently…”* (P06, mother). These strategies highlight how parents used the citation to reinforce responsibility and safer decision-making.

#### 3.2.4 Subtheme 3: perceived impact

Many parents believed the citation had a positive influence on their teens’ driving, increasing caution and awareness. Parents felt the citation served as a wake-up call, helping their teens realize the importance of safe driving. As one mother shared, *“… he definitely learned his lesson. My car is a Lexus SUV, and I’m able to also see his driving patterns on the Lexus app. We have a few hard brakes here and there, but he’s a cautious driver and… I think he takes his time now”* (P11, mother). Others felt the citation heightened their teens’ awareness, particularly of the financial burden associated with citations, which reinforced the seriousness of unsafe driving, *“I think it was a wake-up call for her to understand that financially I would not be able to handle this… If I’m speeding, then something else could happen where I would be responsible for someone’s vehicle…”* (P17, mother). However, not all parents observed lasting change. One mother noted, *“I think she’s still a little bit of a speedy driver”* (P05, mother). Despite this, she, like many others, continued to reinforce safe driving messages. These insights suggest that parents often viewed the citation as an effective motivator for behavior change.

#### 3.2.5 Theme 2: Parents’ experiences with ProjectDRIVE

This theme reflected parents’ perspectives on how ProjectDRIVE helped prompt safe driving practices for their teens. Three subthemes were identified, including parents’ perceived benefits, opportunity for ongoing communication, and objective driving feedback.

#### 3.2.6 Subtheme 1: perceived benefits

Parents consistently described ProjectDRIVE as beneficial, emphasizing that it re-engaged them in their teens’ driving practices and fostered ongoing conversations about safe driving, an involvement they considered essential for lasting behavior change. One mother explained, *“And the reason why I liked [ProjectDRIVE] is because it was holding him accountable, and then we were able to have open discussions about the situations like, ‘Okay, well yeah, that was actually probably the best decision in that type of situation.’ Or ‘Hey, maybe we could make a different decision when doing that, because that could lead to other issues.’”* (P27, mother). Several parents felt the combination of the citation and program helped their teens grasp the seriousness of safe driving responsibilities, *“… the ticket, the accident, plus this program [ProjectDRIVE] put a big spotlight on [safe driving]. Maybe even in their teenage brains, it might’ve sunk in a little bit. Like, ‘oh yeah, I guess this is serious.’”* (P03, father). Parents noted that ProjectDRIVE reassured them about their teen’s ability to drive responsibly after receiving a citation. Many believed the program offered benefits beyond the immediate incident. One mother noted, *“It’d be nice if they had this program for everybody. ….With this program, 6 months of going over everything and in hopes that it’ll stay with him forever…”* (P07, mother).

#### 3.2.7 Subtheme 2: opportunity for ongoing communication

Parents acknowledged that ProjectDRIVE encouraged meaningful conversations that might not have occurred otherwise. Several parents noted that teens were often more engaged in discussions because they were part of a study*: “During those [conversation recordings as part of project] he knew he was being recorded for research purposes so he listened to me and would respond and ask questions”* (P15, mother). Parents also shared that participating in the study helped hold them accountable for discussing driving safety, even with their busy schedules. These structured conversations reminded them that teens continue learning even after licensure. One parent reflected, *“…, it’s still teaching time. She’s not done learning. So that was the point of those conversations is to hopefully know that I continue to support her as a driver”* (P01, mother). Parents in the feedback plus communication training group valued skills like open-ended questions, which reduced defensiveness and deepened conversations. One parent shared, *“What I liked most is it spurred me thinking about how to frame questions or conversations… I think the natural instinct is to just tell kids what to do… I liked how asking open-ended questions, engaging them in the conversation. They get a lot less defensive…. That was good just to have very specific examples of how you could frame a question and generate a conversation”* (P32, mother). Overall, parents indicated that continued conversations about safe driving, especially when supported by communication strategies, helped foster both safer driving behaviors and stronger parent-teen relationships.

#### 3.2.8 Subtheme 3: objective driving feedback

Parents appreciated the real-time in-vehicle feedback and bi-weekly reports, noting these tools increased teens’ awareness and provided clear evidence of driving behaviors. As one mother explained*, “Paying attention to the weekly reports. I think that’s where he can see it right in front of him…I think that’s the results are right there. They can see evidence right in front of them…[that] gets their attention right away”* (P02, mother). Some parents also valued the device and reports as an external source of reinforcement, helping to validate safe driving messages beyond their own guidance, *“The feedback was useful. Once again, if we were not involved, she never would’ve had any feedback other than coming from me. And that’s not always what they desire to hear from parents,”* (P09, mother), and another shared*, “And what I liked of this program, for example, was the fact that my daughter was getting the reports of driving, what she was doing. And I think that that helped a lot, because she was more aware of what she was doing”* (P24, mother). Most parents who received the bi-weekly reports found them helpful for both monitoring their teen’s driving post-citation and sparking ongoing conversations about safe driving, supporting safer long-term habits.

#### 3.2.9 Theme 3: Strategies for strengthening and scaling ProjectDRIVE

This theme captured parents’ recommendations for broadening the reach, effectiveness, and sustainability of ProjectDRIVE. Three subthemes were identified: (1) increasing adoption and stakeholder buy-in, (2) integrating the program into existing systems, and (3) improving program navigation and relational engagement.

#### 3.2.10 Subtheme 1: Increasing adoption and stakeholder buy-in

Parents emphasized that broader implementation of ProjectDRIVE, particularly within juvenile courts, would require compelling evidence, persuasive testimonials, and targeted outreach to key and influential decision-makers. Parents noted that judges influence one another and suggested engaging highly respected “trendsetter” judges to increase court adoption for the program across juvenile courts, and that judges respond to messages about teen safety and community benefit: *“The judge that we had truly seemed to care about teens…I think, how you get to get them to buy in, you speak about the kids and the lives that it could save, and you hit the personal side with the judges”* (P17, mother). Data demonstrating reduced recidivism was considered essential, as one noted, *“They would have to see statistics… about how many teenage drivers’ driving records have improved through the help of your program, have made the roads safer…then that would probably [gain] their interest”* (P12, mother). Parents also viewed testimonials from youth, parents, and judges as especially persuasive: *“Having some testimonials… maybe parent and child but also even having your judge provide some sort of testimonial”* (P17, mother). Several parents also stressed the importance of awareness, noting many learned about ProjectDRIVE only after a traffic citation: *“If the accident did not occur… I would not have learned about the program”* (P13, mother).

#### 3.2.11 Subtheme 2: Integrating the program into existing systems

Parents recommended embedding ProjectDRIVE into existing systems, including juvenile courts, driver’s education, schools, and insurance programs, to broaden access and enhance impact. Most felt that stronger court integration would increase participation and structure, with some advocating for mandatory involvement: *“It would be a great idea for them to make it mandatory, because then the parents would also be involved…I think it’s very useful to have that more of a long-term discussion about driving,… It really is very useful”* (P31, mother). This implies the need for driving programs after teens receive a traffic violation. Many parents also emphasized incorporating the program into driver’s education, ideally before teens start driving independently, to help increase their awareness of risky driving*: “Getting this training before they have an infraction… merge it into driver’s ed”* (P29, mother). Because court-based referrals only include teen drivers with a traffic citation, which excludes many teens, several participants recommended school- or community-based delivery: *“It should be offered in school systems…not every kid is involved in the court system… I think even broadening up the audience that you gear towards would be helpful*” (P13, mother). Others suggested insurance partnerships to encourage participation: *“If you hit the insurance companies… more people would want to do it for the break on insurance”* (P38, mother).

#### 3.2.12 Subtheme 3: Improving program navigation and relational engagement

Parents highlighted the need for streamlined program navigation and stronger relational support. Although they valued access to teen driving data and professional guidance, many found multiple platforms and logins challenging: *“Three or four websites… constantly different usernames… I would’ve been discouraged right off the rip”* (P27, mother). They recommended clearer upfront communication and organizational tools like centralized calendars *“I would’ve liked a calendar… instead of just receiving emails. I get 20,000 emails a day. Sometimes they get lost in the shuffle there”* (P04, mother). Parents also encouraged refining the device and training content to increase flexibility and real-world relevance. Speed alerts were often described as too rigid *“Make that window flexible… but because we could not adjust that at all, we had to turn the beeping off”* (P37, father), and many preferred voice-based cues to uniform beeps. Real-world scenario training was another common recommendation: *“Adding information about different scenarios, with communication with teens…”; “If they ever get into an accident… what information they need to get from the other person…”* (P24, mother). Finally, parents underscored the importance of social and relational support in sustaining engagement. They noted teens’ responsiveness to peers and suggested small-group or teen-only sessions: *“They listen more to other kids… a small group of peers would be a useful cohort to use instead of just individual”* (P15, mother). Parents themselves valued group-based formats that normalized experiences and maintained motivation: *“Zoom calls video with maybe two or three other adults or maybe kids their age that are going through similar thing…and they can hear other people’s feelings”* (P16, mother). Several described the program as creating shared accountability during a challenging time.

## 4 Discussion

This qualitative study explored how parents responded to their teen’s traffic citation, how they experienced the ProjectDRIVE intervention, and how they believed the program could be strengthened and scaled. By situating traffic citations as psychologically salient events within a broader public health context, the findings highlight both the challenges and opportunities in supporting high-risk teen drivers and underscore the central role of parents in shaping teens’ driving behaviors during the early months of independent driving.

Parents frequently described the citation as a stressful yet pivotal event that prompted renewed engagement in their teen’s driving. Although initial reactions often included frustration or concern, many parents shifted toward constructive responses that balanced consequences with emotional support and accountability. This pattern reflects the concept of a “teachable moment,” in which emotionally charged events disrupt routine behaviors and heighten receptivity to guidance. These strategies align with authoritative parenting practices known to promote safer teen driving (16, 34, 35). Consistent with prior research across health domains, including patient care, smoking cessation, and adolescent substance use (11–15, 36), our findings suggest that adverse or stressful events can motivate behavior change when paired with appropriate support. In this context, a traffic citation may serve as a critical catalyst for productive parent-teen dialogue and renewed attention to driving safety.

Parents’ experiences with ProjectDRIVE further demonstrate how structured, multilevel interventions can help families capitalize on this teachable moment. The combination of real-time in-vehicle driving feedback and parent communication training was viewed as particularly beneficial, addressing both behavioral awareness and interpersonal dynamics (25, 26). Parents reported that objective driving data increased teens’ awareness of risky behaviors, reduced reliance on subjective parental judgment, and functioned as a “third-party voice,” reinforcing earlier findings that in-vehicle monitoring systems can enhance risk awareness and reduce unsafe driving (37–40). By increasing teens’ insight into their driving patterns during a period of heightened risk, these tools supported individual behavior change while facilitating parent engagement at the family level.

The communication component of ProjectDRIVE further strengthened parent–teen interactions. Parents described how structured prompts and motivational interviewing–based strategies encouraged more open, less defensive conversations, consistent with prior research on adolescent behavior change and parent communication (19–21, 41). These approaches appeared particularly effective in the emotionally charged period following a citation, when teens may be vulnerable to defensiveness or disengagement. Many parents also emphasized that the program helped sustain their involvement during a developmental stage when parental supervision typically declines (21, 41), reinforcing the importance of continued parental engagement in promoting safe driving behaviors (19, 20, 24).

Parents also offered valuable insights into how ProjectDRIVE could be strengthened and scaled, highlighting the importance of system-level integration. Recommendations to engage influential judges, embed the program within juvenile courts, and use data and testimonials to build credibility align with implementation science principles emphasizing stakeholder buy-in and institutional support (30–32). These suggestions underscore the potential of courts and related systems to serve as public health delivery platforms, particularly for reaching teens at elevated risk. Parents also expressed interest in earlier and broader delivery through driver education programs, schools, and community settings, reflecting a desire to extend prevention beyond court-involved populations (16, 19, 20). Attention to usability, platform consolidation, and relational support further emphasized the need to reduce implementation burden and enhance engagement, consistent with evidence that intervention complexity can limit adoption and sustainability (32).

Across all themes, parents emerged as motivated and influential partners in promoting teen driving safety when provided with timely, practical tools. By centering parents’ lived experiences, this study addresses a critical gap in road safety research, which has often focused on teen behavior or enforcement outcomes without fully examining the family context. Parents occupy a pivotal position at the intersection of adolescent development, legal systems, and technological interventions, making their engagement essential for the success and scalability of post-citation programs.

Taken together, these findings highlight the promise of parent-focused, technology-supported interventions delivered during psychologically salient moments such as traffic citations. Integrating behavioral and technological strategies within existing systems may offer a sustainable, public health–oriented approach to reducing crash risk among high-risk teen drivers. Future efforts should continue to refine, adapt, and scale such interventions to maximize reach, equity, and long-term impact on road safety.

### 4.1 Limitations

This study has several limitations that should be considered when interpreting the findings. First, the sample was predominantly White, female, and college-educated, which may limit the generalizability of results to more diverse populations. Fathers were underrepresented, and the perspectives captured may not fully reflect the experiences or responses of male caregivers. Second, because participants had completed ProjectDRIVE and agreed to a follow-up interview, their responses may reflect higher levels of engagement or social desirability despite assurances that no answers were “right” or “wrong.” Third, interviews were conducted by researchers affiliated with the intervention, which may have introduced response or interviewer bias. Although rigorous qualitative procedures were followed, parents may have provided more favorable feedback about the program. Fourth, as with all retrospective self-reports, parents’ interpretations of their teen’s behavior, both before and after the citation, may be subject to recall bias or influenced by their hopes for improvement. Finally, the generally consistent themes observed across the sample may reflect the perspectives of parents already receptive to structured driving interventions, which may not represent all families of teens who receive traffic citations. Despite these limitations, this study provides insight into how parents may use traffic citations as teachable moments, how objective driving feedback can function as a neutral “third-party” tool to support communication, which factors may contribute to sustained parental engagement after a teen’s citation, and how system-level strategies could inform implementation and future scale-up efforts.

## 5 Conclusion

This study demonstrates that traffic citations can function as psychologically salient teachable moments for families of high-risk teen drivers. With appropriate support, parents reframed these stressful events as opportunities to reinforce accountability, clarify expectations, and promote safer driving behaviors. ProjectDRIVE supported this process by integrating objective, technology-based driving feedback with structured parent communication strategies, helping parents remain engaged during a period when supervision typically declines. Parents reported that these tools increased teens’ awareness of risky behaviors and fostered more constructive, less defensive conversations about driving safety. Parents’ recommendations highlighted the importance of program usability and integration within existing systems, including juvenile courts, schools, and driver education programs. Together, these findings suggest that traffic citations represent a critical yet underutilized opportunity for intervention among high-risk teen drivers. By harmonizing behavioral and technological approaches and engaging parents as key partners, post-citation interventions may help reduce crash risk, improve teen health outcomes, and promote safer communities. Future research should examine how such interventions can be adapted and scaled to reach more diverse populations and advance broader road safety goals.

## Data Availability

The original contributions presented in the study are included in the article/supplementary material, further inquiries can be directed to the corresponding author.
